# Supplementary material for: Patterns of Intron Gain and Loss in Fungi
Source: PLoS Biol. 2004 Nov 30;2(12):e422. doi: 10.1371/journal.pbio.0020422 (PMC532390; doi:10.1371/journal.pbio.0020422)
Supplement: Table S1 — Also available at http://genes.mit.edu/NielsenEtAl/. (4.3 MB ZIP). [file pbio.0020422.st001.zip › NielsenEtAl/html/281.html]

AN1695.1.NCU06039.1.MG08065.1.FG07525.1


```
 CLUSTAL W (1.82) Multiple Sequence Alignments - Introns Inserted


Sequence 1: NCU06039.1	592 aa
Sequence 2: MG08065.1	484 aa
Sequence 3: FG07525.1	494 aa
Sequence 4: AN1695.1	609 aa
Alignment Length: 652 aa
Number Identitical Residues: 253 aa
Alignment Score (without introns) 12240


MG08065.1 	---------------------------------------------------------MNS
NCU06039.1	MNQQSQQYQPARRPSAYASHGDELHMSAH------QAHLSSPREYASSGAPHIKLEQMPS
FG07525.1 	---------------------------------------MSPQEYENP--PQIKFESSPS
AN1695.1  	MTNPAQPFD-SRRSTIYNRQPEELHIPAGNSTSGGHPRQPMSHEYPTSPDTHLPSINIHS
          	 .. :.  . :  .:      ..   .: .:::.. .    . .  ..  .     .  *

MG08065.1 	S-----------------GVPNILRPGG~-------------------------------
NCU06039.1	SPPNMQSYQ------SAGAVPNVLQPGG~MSSRPPVMQSNTAPTLPTMQQSADYKSHGYE
FG07525.1 	NSHYRNP--------SNTSVPNVLQPAG1-------------------------------
AN1695.1  	SSSQSNQYGGASGGVTGGTLPGSLQPGN~SANRPPTVSMNTAPSTIPTLPHLSTQIQQQP
          	..   .   .::.. :   :*. *:*..  :. ..  . .::.:  .     . .     

MG08065.1 	------------------------------------------------SQRNVSNTPLGL
NCU06039.1	RSSPATLASNSYDGGNSPYSAYNPTPPGGSSSSQYMSPASTNKYAAASSSRNVSNTPLGL
FG07525.1 	------------------------------------------------SQRNISNTPLGL
AN1695.1  	QPQPQSTTPRSNAANSHGHSRSSPAG-------YRPHGSSPNAAFQPPTPQGAKYSPLGL
          	 ... : :. :  ...   :  ..:             ::..    ..: :. . :****

MG08065.1 	ADIRPRADSSLSDG--IPGSAAYDVANTQSGTSNYMAPWALYAFDWCKYPPQGNG---AG
NCU06039.1	SDIRPRADSTLSDGM-LPGSQAYELANPQSRTSNYMAPWATYSFDWCKWRPSANS---AG
FG07525.1 	ADIRPRADSSMSDG--TGAAMAYDANNAQPGTSNYLAPWAIYAFDWCKWPAQGNS---AG
AN1695.1  	ADIRPSGDLLGDHATNAGAAASKSVENQVPTNSNYITPWPIYAVDWCKWPISGNPGSFGG
          	:**** .*   ... .  .: : .  *  . .***::**. *:.****:  ..* .: .*

MG08065.1 	KLAIGSYLEDGHNF0IQILDTQVVPTPPEAYSQGGSRTCLEFTKVAEATHSYPVTRLLWE
NCU06039.1	KLAIGSYLEDGHNY~IQILEANLTQTPPDVYVPGTSKYSMDFTRTAEATHSYPVTRLLWE
FG07525.1 	KLAVGSYLEDGHNF0IQILDSHITPTPQDIYTPGSSKFSLEFTKAAEATHSYPVTRLLWE
AN1695.1  	KIALGSYLEDHHNY0IQILDTHLAYPDPDTPDASAGELKLEYVKSAEATHSYPVTRILWE
          	*:*:****** **: ****::::. .  :    . ..  :::.: ***********:***

MG08065.1 	PHSPNSQKQSTDLLATSGDHLRLWSLPSD--ASSSMSNNINRSSRDPAPPTKLTPLALLS
NCU06039.1	P--PSSQKQSTDLLATSGDHLRLWSLPADPVVPTSGSSITSRSGRD-APITKLTPLALLS
FG07525.1 	P--PSSQKQSTDLLATSGDHLRLWSLPSE--NPSTPSGTIGR--RD-NVTTKLTPLALLS
AN1695.1  	P--PSSQKQSTDLLATSGDHLRLWSLPNSQPQHSSNSITRPSGQRD-MPAAKLSPLALLS
          	*  *.********************** .    :: *     . **    :**:******

MG08065.1 	NSKTPDHTAPLTSLDWNQVSQNLIITSSIDTTCTIWDIPSLTAKTQLIAHDKEVYDVRFC
NCU06039.1	NSKTPDHTAPLTSLDWNTVQPSLIITSSIDTTCTIWDIPSLTAKTQLIAHDKEVYDVRFC
FG07525.1 	NSKTPDHTAPLTSLDWNTVSPSLIITSSIDTTCTIWDIPSLTAKTQLIAHDKEVYDVRFC
AN1695.1  	NSKSPEHTAPITSLDWNTISPSLIITSSIDTTCTIWDIPTLTAKTQLIAHDKEVYDVRFC
          	***:*:****:****** :. .*****************:********************

MG08065.1 	ANSVDVFVSCGQDGSVRMFDLRSLEHSTIIYEPTGKDEKD1PSGGRISPTLAQQTMSNPP
NCU06039.1	ANSVDVFVSCGQDGSVRMFDLRSLEHSTIIYEPTGKEERD1ANGGRISPTLAQQTMSHPP
FG07525.1 	AKSVDVFVSCGQDGSVRMFDLRSLEHSTIIYEPTGKEERD1PNGGRVSPTLAQQTMSNPP
AN1695.1  	ANSVDVFVSCGADGSVRMFDLRSLEHSTIIYEPTEKNEKL1MSPGNGSP--SAPTTTWPP
          	*:********* ********************** *:*:   . *. **  :  * : **

MG08065.1 	PLLRLAT~SPHDTHLLATFAQDSNVIRILDVRQPGQALLELRGHGGSLNCIEWSPLRRGT
NCU06039.1	PLLRLAT~SPHDQHLLATFAMDSNVIRILDVRQPGQALLELRGHGGSLNCIEWSPTRRGT
FG07525.1 	PLLRLAT~SPHDTHLLATFAQDSNAIRILDVRQPGQALLELRGHAGNINSIEWSPHRRGM
AN1695.1  	PLLRISA1SPHDAHLLATFSQDSNIVRVLDVRQPGQALLELKGHSAPLNTVEWSPNRRGV
          	****::: **** ******: *** :*:*************:**.. :* :**** *** 

MG08065.1 	LASGADDCQVLIWDLLQNQSAAPPLNGAPQPDNV-----RSPVASWQCDYEIGNLGWTPQ
NCU06039.1	LASGGDDCQVLIWDLLNQPSGLTPAG---QPDNV-----RSPVASWQCDYEVGNLGWVPP
FG07525.1 	LASGGDDCQVLLWDIYNSGQ---PINGAPQQDNP-----RSPVASWQCDYEIGNLGWVPQ
AN1695.1  	LASGADDCLVLLWDLINQHNTTPVPPGVHNPGAPSTTTERGPAAAWQCDYEISNISWSPQ
          	****.*** **:**: :. .  .   .  : .  ::::.*.*.*:******:.*:.* * 

MG08065.1 	LANSENG---EWLGVSAGRGIWGLK0TIDRSFGGAEPSSLHRMLGDWMAYIGPANRV
NCU06039.1	MGNGEYG---DWLGVSAGRGVWGVR~F------------------------------
FG07525.1 	LPNSEYG---EWLGIGAGRGIWGTR0VS-KAWAGREGGSIERIFHWRLALE------
AN1695.1  	GGTTSAGHPRDWLGVCGGRGVWGVA~L------------------------------
          	  . . * . :***: .***:**
```
